# Cortical Organoid-on-a-Chip with Physiological Hypoxia for Investigating Tanshinone IIA-Induced Neural Differentiation

**DOI:** 10.34133/research.0273

**Published:** 2023-11-22

**Authors:** Yue Zhi, Yujuan Zhu, Jinglin Wang, Junqi Zhao, Yuanjin Zhao

**Affiliations:** ^1^ Department of Rheumatology and Immunology, Nanjing Drum Tower Hospital, Clinical Medical College of Traditional Chinese and Western Medicine, Nanjing University of Chinese Medicine, Nanjing, 210023, China.; ^2^ State Key Laboratory of Bioelectronics, School of Biological Science and Medical Engineering, Southeast University, Nanjing, 210096, China.; ^3^ Shenzhen Research Institute, Southeast University, Shenzhen, 518038, China.

## Abstract

Cortical organoids represent cutting-edge models for mimic human brain development during the early and even middle stage of pregnancy, while they often fail to recreate the complex microenvironmental factors, such as physiological hypoxia. Herein, to recapitulate fetal brain development, we propose a novel cortical organoid-on-a-chip with physiological hypoxia and further explore the effects of tanshinone IIA (Tan IIA) in neural differentiation. The microfluidic chip was designed with a micropillar array for the controlled and efficient generation of cortical organoids. With low oxygen, the generated cortical organoids could recapitulate key aspects of early-gestational human brain development. Compared to organoids in normoxic culturing condition, the promoted neurogenesis, synaptogenesis and neuronal maturation were observed in the present microsystem, suggesting the significance of physiological hypoxia in cortical development. Based on this model, we have found that Chinese herbal drug Tan IIA could promote neural differentiation and maturation, indicating its potential therapeutic effects on neurodevelopmental disorders as well as congenital neuropsychiatric diseases. These results indicate that the proposed biomimetic cortical organoid-on-a-chip model with physiological hypoxia can offer a promising platform to simulate prenatal environment, explore brain development, and screen natural neuroactive components.

## Introduction

The cerebral cortex is the integration and execution center of the human central nervous system (CNS), accounting for more than half of the brain volume. It is hypothesized to be accountable for the neuronal computations behind complicated phenomena such as cognition, language, concentration, and voluntary movement. Alterations in the cortex that result from abnormal neural differentiation are correlated with a variety of developmental disorders, including mild learning disabilities and even severe intellectual disability [1–3]. Thus, a full understanding of the complex biological process and mechanism of neural differentiation may help us apply therapeutic strategies against diseases of the neural system, such as neurodevelopmental and neurodegenerative diseases [4–6]. Generally, single-cell models have provided insights into the differentiation of the human brain but do not capture its full complexity [7]. Cortical organoids, as a novel 3-dimensional (3D) model that encapsulates key structural and functional features of the fetal early-gestational brain, have promising applications in the study of brain development and disease [8–10]. Despite great progress, current cortical organoid systems often fail to recapitulate the complex microenvironment during embryonic development, such as physiological hypoxia. Studies have shown that oxygen level is critical in the development and function of vital tissues and organs, especially for neuronal cells [11–13]. Physiological hypoxia is involved in the regulation of various neuronal cells in the CNS and plays a neuroprotective role after injury, which may be a potential method for the treatment of nervous system diseases. However, few studies have been performed to systematically investigate the impacts of physiological hypoxia on the neural differentiation of embryos in vitro [14]. Therefore, it is highly desirable to develop the human cortical organoid system with physiological hypoxia.

**Fig. 1.**
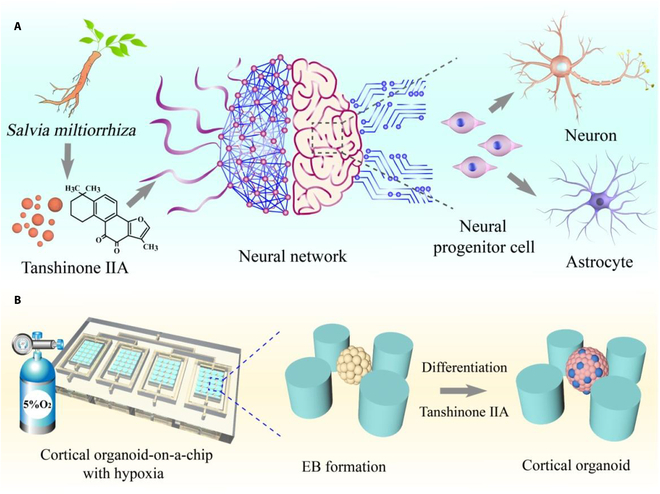
Illustration of the microsystem with physiological hypoxia, allowing to probe the effects of Tan IIA in neurodevelopment. (A) Tan IIA promotes the formation and function of neural networks, which is reflected in the differentiation and maturation of various types of cells. (B) Scheme showing human cortical organoids exposed to low oxygen in a hypoxic incubator. The overall structure of the microsystem and the enlarged image of the internal details show the corticogenesis process.

Herein, following the process of embryonic development, a novel cortical organoid-on-a-chip with physiological hypoxia is proposed, which allows us to investigate the impact of tanshinone IIA (Tan IIA) on the early neurodevelopment of the fetal cortex, as schemed in Fig. 1. As an emerging second-generation organoid-on-a-chip, it was first proposed in 2019, which allows researchers to not only model physiological and disease processes on-chip but also monitor and track these processes in real time [15–19]. However, few studies utilized the technology to investigate the effects of physiological hypoxia on the neural differentiation trajectory during early human brain development. In another aspect, more and more attention has been paid to the development and utilization of traditional Chinese medicine resources worldwide as the traditional Chinese herbal materials and their extracts have remarkable effectiveness in the prevention and treatment of diseases [20–23]. In particular, many Chinese herbs, such as lipophilic Tan IIA from *Salvia miltiorrhiza*, are found to have both neurogenic and neuroprotective effects, with potential ameliorative and therapeutic properties for neurodevelopmental disorders, nerve damage, or other related diseases [24–26]. However, due to the lack of an efficient platform, the complicated mechanisms of Tan IIA on neurogenesis, neuronal differentiation, and neural networks have not been demonstrated yet.

In this paper, we constructed the biomimetic cortical organoid-on-a-chip microsystemand investigated the efficacy of Tan IIA on early fetal brain progression. To our knowledge, this is the first report to established a highly bionic brain organoid-on-a-chip model in vitro that recapitulates intracerebral hypoxic microenvironment. The microfluidic chip was composed of micropillar arrays, which allows for the controlled and efficient generation of cortical organoids derived from human induced pluripotent stem cells (hiPSCs). With physiological hypoxia, cortical organoids displayed notable variations in neural differentiation and maturation in comparison to cortical organoids in conventional culture systems, as confirmed by flow cytometry, immunofluorescence analysis, and quantitative real-time polymerase chain reaction (qRT-PCR). These demonstrated that our system is promising for studying the effects of hypoxia on human cell differentiation and morphogenesis in tissue environments. On this basis, we evaluated the efficacy of Tan IIA on cortical development under a near-physiological condition and found that the Tan IIA could promote neuronal cell differentiation and neural network formation. These results implied that the established model can be utilized for probing neurodevelopmental conditions of early gestational offspring. Due to the similarity to the in vivo hypoxic environment, our microsystem offers a promising alternative for regenerative medicine, traditional Chinese medicine testing, and developmental biology.

## Results

### Construction of the microsystem

**Fig. 2.**
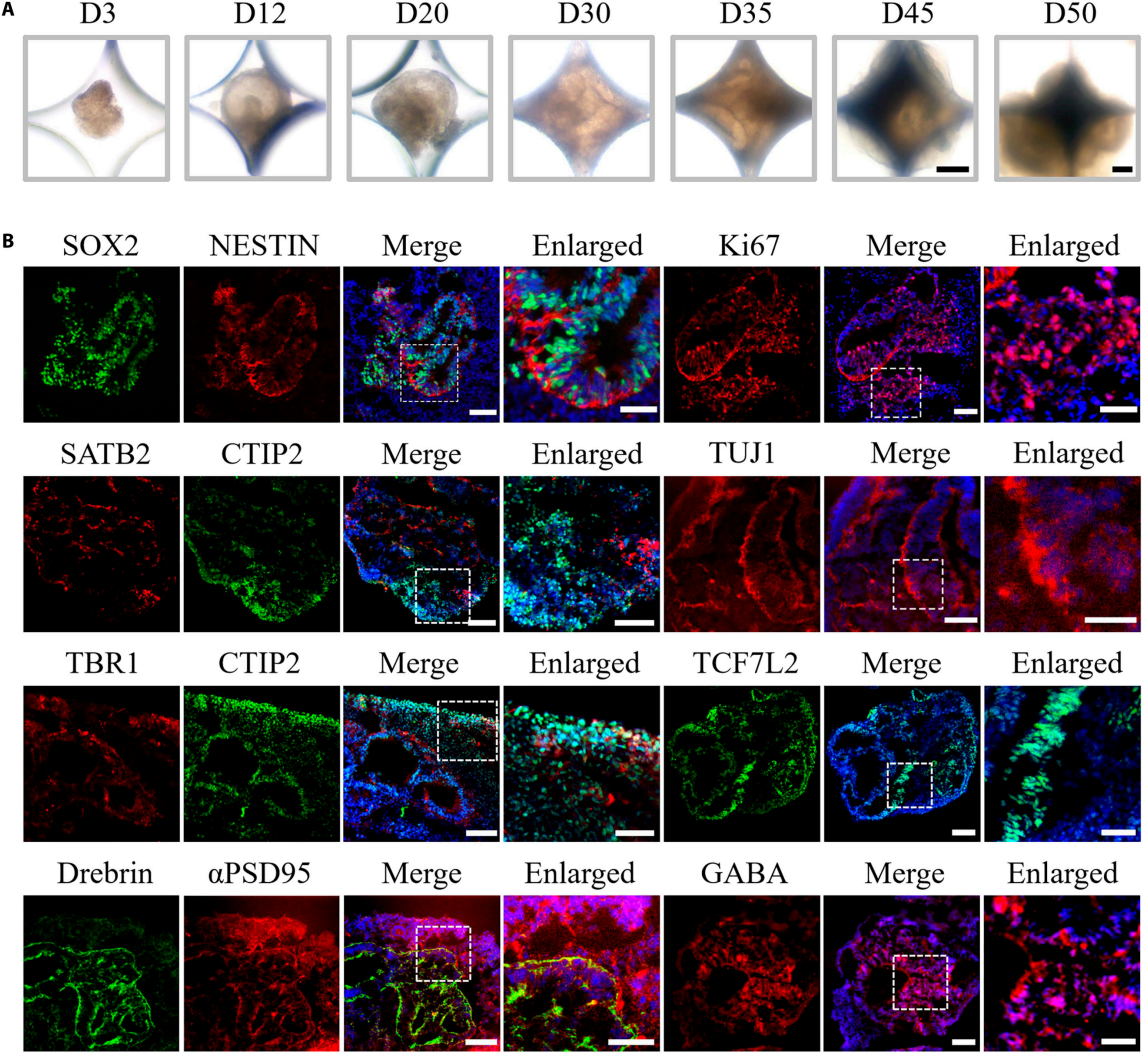
Formation and validation of the human cortical organoid under hypoxic conditions. (A) Representative bright-field pictures of the cortical organoids generated on the chip within the hypoxic environment. Scale bars, 500 μm. (B) The immunofluorescence images of NPCs (SOX2 and NESTIN), proliferative marker Ki67, cortical layer (TBR1, SATB2, and CTIP2), differentiated neurons (TUJ1), postsynapse (αPSD95 and drebrin), Wnt signaling pathway (TCF7L2), and inhibitory neurons (GABA) within 40-d cortical organoids. Scale bars, 100 μm (in “Merge”) and 50 μm (in “Enlarged”).

In typical experiments, a microfluidic system with micropillar arrays was devised and built (Figs. S1 and S2), permitting the regulated generation of embryoid bodies (EBs), cellular differentiation, and maturation. Also, the chip consisted of 4 independent chambers, facilitating the efficient screening of drug candidates, thereby reducing the variation between groups due to the complex operations. In particular, hiPSCs formed EBs that possessed coincident shape and dimensions between micropillars. Subsequently, EBs were cultured in medium containing the corresponding factors for induction and differentiation on the chip, which produced an optimal condition for the development of neuroectoderm. The shape and space of the micropillars were finely designed, which prevented nearby spheroids from fusing together during the incubation process and conquered the shortcomings of conventional culture systems. To replicate the in vivo hypoxic environment during human corticogenesis, we used a hypoxic incubator to expose cortical organoids at day 1 to low-oxygen conditions (5% O_2_) for 30 and 50 d. Ultimately, neuroectodermal spheroids were shown to develop into almost millimeter-sized cortical organoids after being incubated for another 1 to 7 weeks, suggesting the feasibility of physiological hypoxia for organoids culture (Fig. 2A). As a result, the cortical organoid-on-a-chip technology we proposed offered a straightforward and reliable platform for controlling the formation of EBs, which is critical for minimizing the variations in size and morphology of cortical organoids.

### Characterization of cortical organoids on chip

During embryonic development, fresh neurons and glial cells are the differentiated progeny of neural stem cells and progenitor cells, which eventually develop into a regionalized brain; this process is known as neurogenesis [27]. Thus, we explored whether neocortical neuron populations existed within the human cortical organoids. qRT-PCR was adopted to assess various pluripotency and neural lineages within cortical organoids in order to ascertain the efficiency of preliminary neural induction (Fig. S3). In comparison with the control (day 0 EBs), pluripotency markers OCT4 and NANOG were predictably down-regulated at day 15, while the forebrain marker PAX6 was markedly elevated, suggesting efficient neuroepithelial induction. These data displayed the capacity of cortical organoids to achieve differentiation and formation at early stage under hypoxic conditions. Subsequently, cortical organoids were incubated within hypoxic environments for 40 d, and immunostaining images revealed a amount of neural progenitor cells (NPCs) and differentiated neuron as evidenced by the expression of SOX2, NESTIN, and TUJ1 (Fig. 2B). The image displayed a high density of neonatal neurons located around NPCs, confirming that a clear neural identity of the cortical organoids had been created. According to Fig. 2B, the proliferative marker Ki67 was highly expressed in cortical organoids. It was concluded that the system we constructed allowed NPCs to differentiate effectively.

Highly organized laminar structure is the distinctive characteristic of the cerebral cortex, which is crucial for higher cognitive functions [28]. The cortical layer markers COUP-TF-interacting protein 2 (CTIP2), T-box brain protein 1 (TBR1), and Special AT-rich sequence-binding protein 2 (SATB2) were investigated to determine whether there were signs of a clearly stratified cortical architecture in cortical organoids (Fig. 2B). CTIP2+ early neurons were observed to be around preplate TBR1+ neurons by immunohistochemical analysis, and the separation of SATB2+ (superficial layer) and CTIP2+ (deep layer) neurons become prominent (Fig. 2B), implying that the cortical plate has formed. In addition, after 40 d of differentiation, the inhibitory neuron subtype (gamma-aminobutyric acid [GABA]) was discovered in cortical organoid sections. Immunostaining for the Wnt-related marker transcription factor 7 like 2 (TCF7L2), postsynaptic markers α-postsynaptic density protein 95 (αPSD95) and drebrin showed high expression, suggesting synaptic connectivity in the organoids (Fig. 2B). These data showed that cortical organoids generate the overall cytoarchitecture of the developing embryonic cortex. Thus, the feasibility and efficiency of cortical differentiation on the chip under physiological hypoxic conditions were verified.

### Promoted differentiation of cortical organoids under hypoxia

**Fig. 3.**
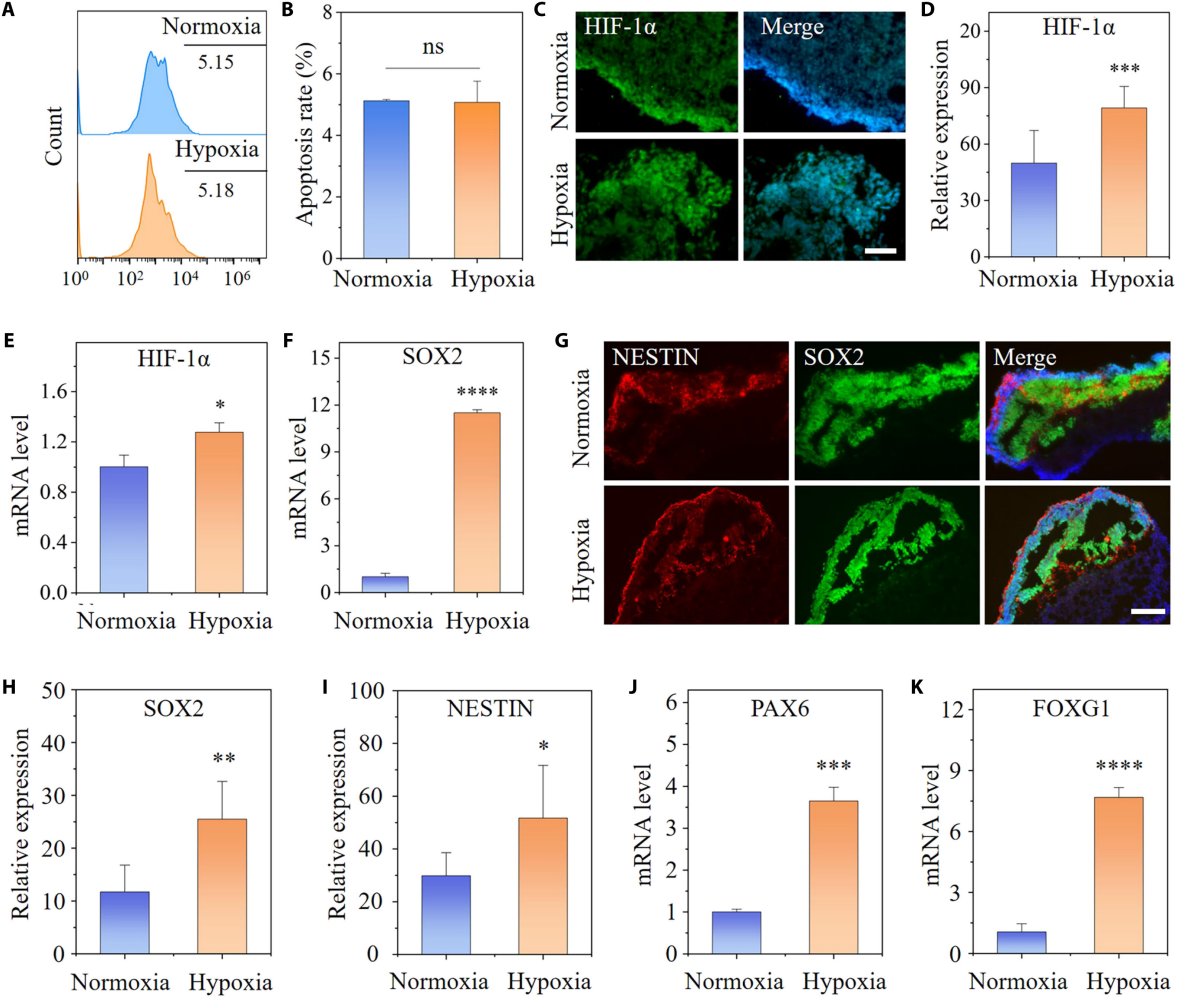
Cellular responses to hypoxia in cortical organoids. (A and B) Fluorescence-activated cell sorting analysis and quantitative results of apoptosis in hypoxia- and normoxia-exposed cortical organoids at day 30 (*n* = 3). (C and D) Representative immunostaining and quantitative analysis of HIF-1α in cortical organoids exposed for 30 d to 5% O_2_ versus 21% O_2_ (*n* = 6). (E and F) Expression of HIF-1α and SOX2 at the mRNA level in cortical organoids under normoxic or hypoxic conditions at day 30. (G) Immunofluorescence images for SOX2- and NESTIN-positive areas in cortical organoids under low oxygen for 30 d. (H and I) The fluorescence intensity of SOX2 and NESTIN was quantified at day 30 (*n* = 6). (J and K) mRNA Expression of PAX6 and FOXG1 in cortical organoids under normoxia or hypoxia at day 30 was identified by qRT-PCR (*n* = 3). All data are the means of at least 3 replicates ± SD. The data were analyzed using the Student *t* test (ns, not significant; *P < 0.05; **P < 0.01; ***P < 0.001; ****P < 0.0001). Scale bars, 50 μm (C) and 100 μm (G).

To get a full picture of the state of differentiation in cortical organoids within our system, we next compared cellular variations in cortical brain organoids under the hypoxic environment versus the conventional normoxic environment. First, we detected the cellular activity of organoids by using flow cytometry. The level of cell death at day 30 did not reveal an evident distinction between the hypoxia and the normoxia group (21% O_2_) (Fig. 3A and B), suggesting that a hypoxia-like response was induced without massive cell death and that subsequent functional validation could be performed. Hypoxia-inducible factor (HIF)-1α regulates many downstream responses to hypoxia in the CNS [14,29]. Quantitative PCR analysis revealed that the organoids under hypoxia possessed a higher expression of HIF-1α than the normoxia group at both earlier (day 30) and later (day 50) developmental stages (Fig. 3E and Fig. S5). Similarly, immunofluorescence staining demonstrated that HIF-1α was predictably localized in nuclei, and the quantitative analysis of HIF-1α exhibited a similar expression trend (Fig. 3C and D).

As cell division in NPCs is the starting point for neuronal formation, we next evaluated the proliferation of NPCs in hypoxia-exposed cortical organoids. qRT-PCR revealed that, compared with that in the normoxia group, a higher expression level of SOX2 was detected in the hypoxia group both at days 30 and 50 (Fig. 3F and S5). To further validate the changes of NPCs at the protein level due to hypoxia, immunofluorescence staining for SOX2 and NESTIN has been applied. It indicated their greater expression levels in hypoxia-exposed organoids at day 30 compared with organoids cultured under normoxic (21% O_2_) conditions (Fig. 3G), which was validated by quantitative assessment of fluorescence intensity in SOX2+ and NESTIN+ cells (Fig. 3H and I). In line with the phenomenon above, even after being exposed to hypoxia for 50 d, the cortical organoids still retained stronger NPCs expression (Fig. S6A and C). The observation of a dramatic rise in NPCs at the mRNA and protein levels suggested that physiological hypoxia exposure promotes the proliferation of NPCs and triggers the enhanced differentiation of hiPSCs into NPCs, implying that hypoxia appear to be critical in the development of NPCs.

As cerebral development progressed, we investigated the impact of physiological hypoxia on forebrain differentiation within cortical organoids. Gene expression in specific cerebral regions has been assessed in cortical organoids cultured in a low-oxygen environment for 30 and 50 d. qRT-PCR revealed that the level of the PAX6 and FOXG1 (forebrain markers) were substantially elevated in the group exposed to hypoxia (Fig. 3J and K and Fig. S5). These findings indicated that physiological hypoxia may contribute to forebrain development in cortical organoids.

### Enhanced maturation of cortical organoids under hypoxia

**Fig. 4.**
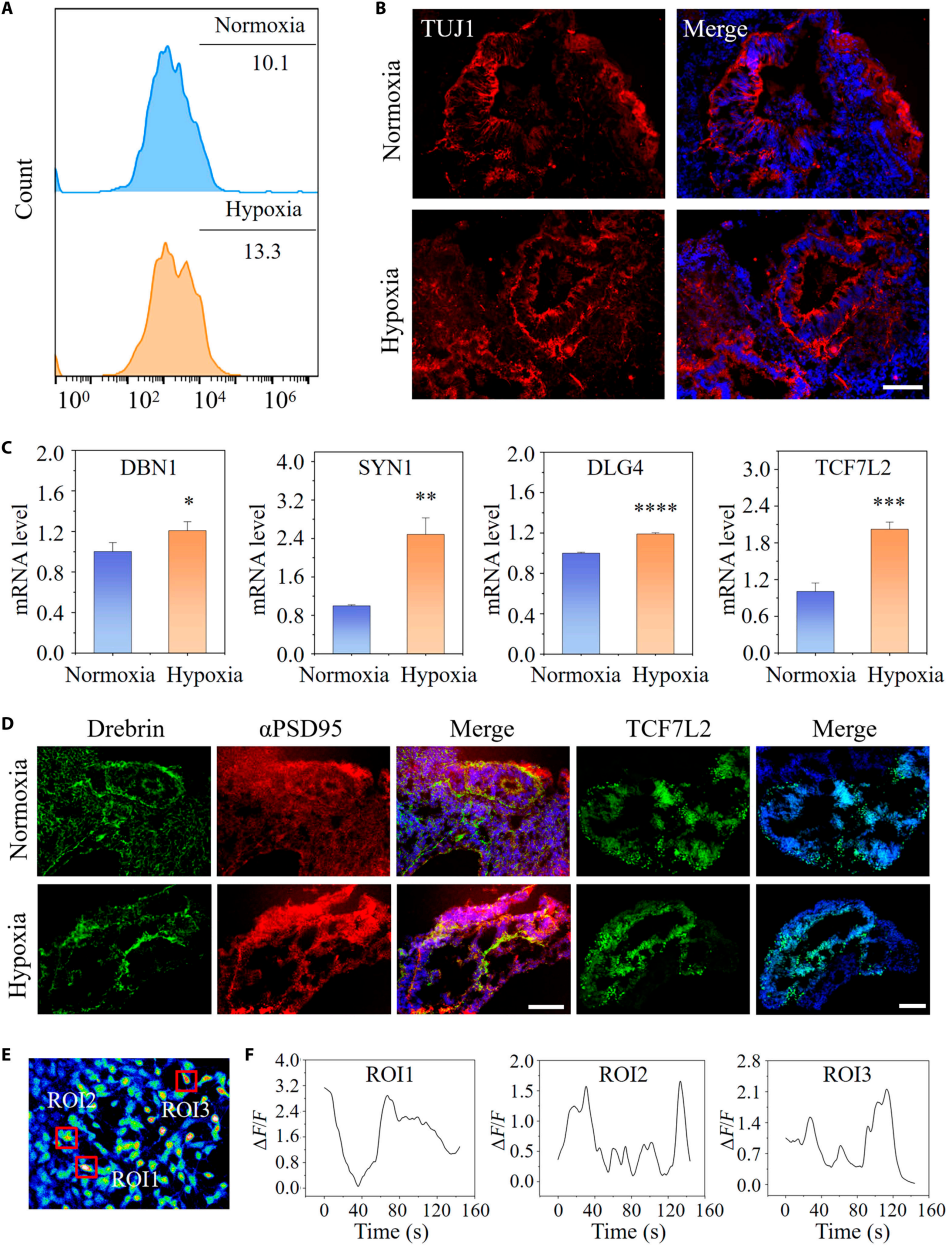
Neuron differentiation and neural network formation under hypoxic conditions. (A) Fluorescence-activated cell sorting analysis of TUJ1+ in hypoxia- and normoxia-exposed cortical organoids at day 30. (B) TUJ1+ cells in cortical organoids treated with hypoxia or normoxia at day 30 were identified by immunohistochemical analysis. (C) The relative mRNA expression of synaptic relative genes at day 50 (SYN1, DBN1, DLG4, and TCF7L2) was determined by qRT-PCR (*n* = 3). (D) Immunostaining images of postsynaptic markers (drebrin and αPSD95) and Wnt-related marker (TCF7L2) with the treatment of hypoxia or normoxia at day 50 are shown. (E and F) Calcium imaging of hypoxia-treated cortical organoids at day 30 as assessed using fluorescence alterations. Δ*F*/*F*: relative concentration of Ca^2+^. Red boxes designated ROIs (regions of interest). All data are the means of at least 3 replicates ± SD. The data were analyzed using the Student *t* test (ns, not significant; *P < 0.05; **P < 0.01; ***P < 0.001; ****P < 0.0001). Scale bars, 100 μm.

As it has been demonstrated, neural activity is essential for regulating and sculpting the complex circuitry of the nervous system; the formation of neural networks is crucial to cortical development [30]. Thus, the cortical organoids exposed to hypoxia and normoxia were examined to determine whether our organoids culture system produced functionally active neurons and neuronal networks. We explored the the level of neuronal marker TUJ1 in the normoxia and hypoxia groups by qRT-PCR, flow cytometry, and immunofluorescence assays. According to qRT-PCR outcomes, the expression level of the neuron-related gene TUJ1 was dramatically enhanced in hypoxia-exposed cortical organoids at day 30 (Fig. S4). With prolonged hypoxia exposure for 50 d, the cortical organoids exhibited a higher expression of TUJ1 compared to the normoxia group, confirming the increased neuronal differentiation in the hypoxia group (Fig. S5). Flow cytometry was conducted to monitor the proportion of TUJ1+ cells in the cortical organoids at day 30. In comparison to the normoxic group, flow cytometry demonstrated more differentiated neurons in hypoxia-exposed cortical organoids at day 30 (Fig. 4A). Consistently, immunofluorescence staining showed that cortical organoids treated with low oxygen possessed more neurons, as evidenced by a greater number of TUJ1+ cells in comparison to that in the control (Fig. 4B). Taken together, these results indicate that hypoxia could promote and direct NPCs to differentiate into neuron. We further compared the level of astrocytes within organoid under hypoxic versus normoxic environment at day 50. As shown in Figs. S5 and S6A and B, the mRNA and protein levels of glial fibrillary acidic protein (GFAP) both surged in hypoxia-cultured organoids, indicating that enhanced proliferation and differentiation of astrocytes stimulated by physiological hypoxia may have a role in maintaining of neuronal networks and cerebral microcirculation.

Advanced brain functions, such as complex behavioral tasks and cognition such as memory, are dependent on accurate connection and efficient interaction between individual neurons within neural network [31]. The evolution of brain circuitry and the regulation of animal behavior are both mediated by synaptic activity, which is at the core of all neuronal functions [32,33]. Next, we detected the expression of mRNA and proteins associated with synapses in cortical organoids at day 50. The postsynaptic density (αPSD95) is a cytoskeletal specialization that plays a role in the anchoring of neurotransmitter receptors as well as the regulation of postsynaptic neurons’ responses to synaptic stimulation. Drebrin, an actin-binding protein, is essential for dendritic spine development and synaptic plasticity. We therefore analyzed the expression of relevant presynaptic and postsynaptic genes and observed the up-regulation of these in hypoxia-exposed ones at the mRNA level (Fig. 4C). Subsequently, we stained cortical organoid slices with postsynaptic marker αPSD95 and drebrin at day 50 to detect synaptic puncta. The proportion of synaptic puncta in cortical organoids was elevated notably within the hypoxia group in contrast to the normoxia group (Fig. 4D and Fig. S7). TCF7L2 is a vital Wnt/-catenin signaling mediator in a variety of cellular functions spanning from early development to mature tissue homeostasis. TCF7L2 expression in neurons throughout early development is essential for the complex characteristic, as well as neuronal gene expression and synaptic transmission. As shown in Fig. 4D and Fig. S7, immunohistochemistry analysis and quantification revealed that hypoxia, in comparison to normoxia, dramatically accelerated the development of the cells with TCF7L2+ nuclei. Consistently, cortical organoids treated with hypoxia exhibited increased expression of TCF7L2 at the mRNA level (Fig. 4C). All of the aforementioned findings demonstrated that hypoxia treatment enhances neurogenesis in cortical organoids, along with promoted neuron differentiation, reinforced functional synaptic connections, and accelerated neural maturation. To explore whether cortical organoids formed functional connections, we utilized calcium imaging, which has emerged as the technique of choice for capturing neuronal ensemble dynamics. Neurons inside cortical organoids differentiated for 50 d already displayed calcium transients, confirming the generation of functional neural junctions (Fig. 4E and F and Movie S1). These outcomes exhibited dynamic neural migration, implying that neural function may be efficiently formed inside cortical organoids based on the proposed microfluidic device under hypoxic settings.

### The impact of Tan IIA on neural differentiation

Based on this reliable model of cortical development, we further evaluated the effects of drugs (such as Tan IIA) in neuronal differentiation in the engineered cortical organoids. First, cortical organoids on the chip were treated with Tan IIA at different concentrations. Cortical organoids which exposed to hypoxic conditions for 20 d were incubated with 0.1, 1, or 10 μM Tan IIA for 72 h and then maintained for 1 week without Tan IIA. Figure S8 shows the flow cytometry viability analysis of cortical organoids treated with Tan IIA at 4 different concentrations in separate culture chambers. The quantitative analysis suggested that more than 95% of the cells in organoids incubated with 0.1 and 1 μM Tan IIA are alive following the 3-d culture period inside the microfluidic chip. Conversely, cells treated with 10 μM Tan IIA demonstrated cytotoxic effects. Moreover, in order to precisely observe the amount of neurons after the drug testing, flow cytometry was performed and the results showed that the cortical organoids treated with 1 μM Tan IIA exhibited the highest proportion of TUJ1 (Fig. S9). The concentration of Tan IIA was thus selected at 1 μM for the following validation tests.

**Fig. 5.**
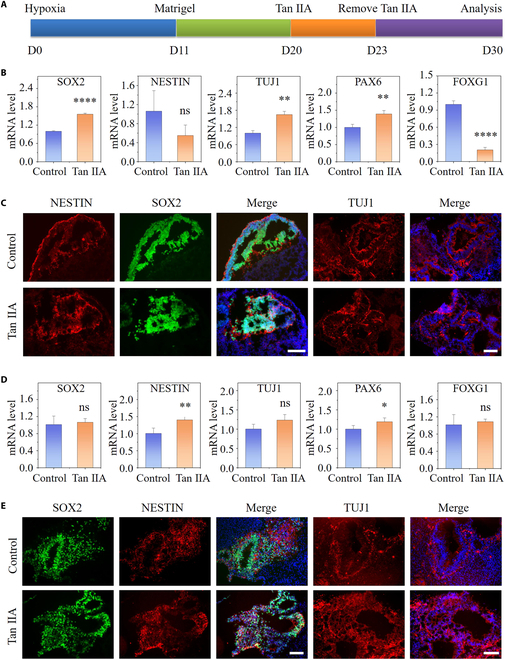
Tan IIA-induced neuronal differentiation and corticogenesisin organoids under hypoxia. (A) Experimental flow chart of treatment of cortial organoid with Tan IIA at an earlier developmental stage. (B and D) Expression of neural genes at the mRNA level in cortical organoids with the presence or absence of Tan IIA at days 30 (B) and 50 (D) (*n* = 3). (C and E) Immunostaining images of SOX2, NESTIN, and TUJ1 with treatment of Tan IIA or not at days 30 (C) and 50 (E) are shown. All data are the means of at least 3 replicates ± SD. The data were analyzed using the Student *t* test (ns, not significant; *P < 0.05; **P < 0.01; ****P < 0.0001). Scale bars, 100 μm.

**Fig. 6.**
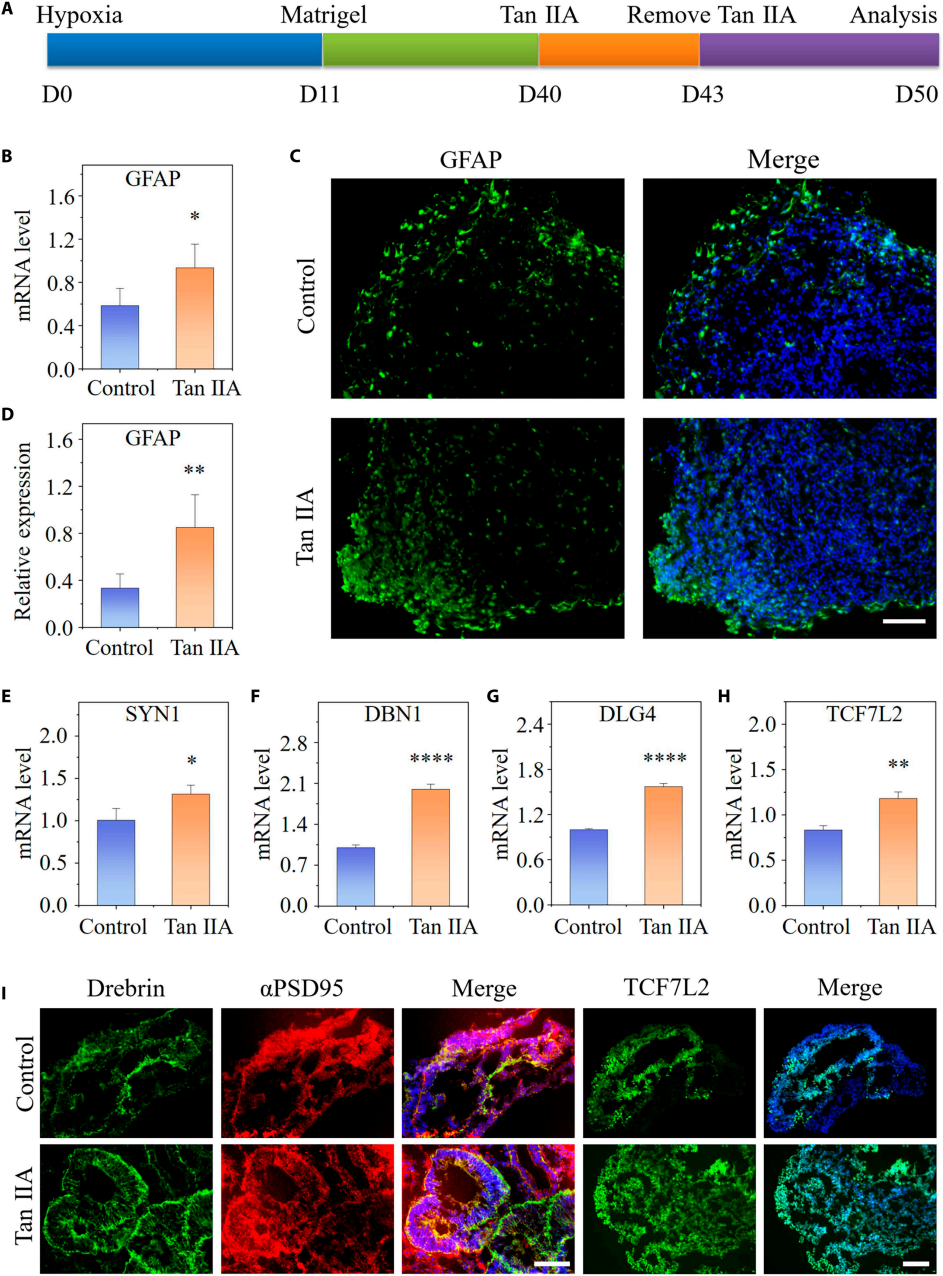
Tan IIA-induced functional differentiation and neural network maturation in cortical organoids under hypoxia. (A) Experimental flow chart of treatment of cortial organoid with Tan IIA at a later developmental stage. (B) Expression of GFAP by qRT-PCR within cortical organoids under the treatment of Tan IIA or not at day 50 (*n* = 3). (C and D) Immunofluorescent staining for GFAP and quantifications for the expression of GFAP in cortical organoids with and without Tan IIA treatment at day 50 (*n* = 6). (E to H) Comparison of relative mRNA expression in synaptic relative genes within 50-d cortical organoids with or without Tan IIA treatment by qRT-PCR (*n* = 3). (I) Immunohistochemical staining for αPSD95, drebrin, and TCF7L2 in cortical organoids at day 50. All data are the means of at least 3 replicates ± SD. The data were analyzed using the Student *t* test (*P < 0.05; **P < 0.01; ****P < 0.0001). Scale bars, 100 μm.

To evaluate the efficacy of Tan IIA on distinct differentiation stages, we dissected organoids at earlier (day 30) and later (day 50) developmental stages in our system. Specifically, cortical organoids exposed to hypoxia for 20 and 40 d were treated with Tan IIA for 3 d, followed by continued maintenance for 1 week under the condition of hypoxia without Tan IIA, and validation tests were conducted at the end (Figs. 5A and 6A). The cortical organoids cultured in hypoxic environments without Tan IIA acted as the control group. Alterations in NPCs (NESTIN and SOX2) and neurons (TUJ1) in response to Tan IIA were further observed in the pre- and postdevelopmental stages of cortical organoids. As shown in Fig. 5C and E, Tan IIA raised the number of NPCs and neuronal cells in cortical organoids at days 30 and 50, as measured by elevated mRNA and protein expression of SOX2 and TUJ1, as evaluated by qRT-PCR and immunostaining, respectively. The tendency of quantitative fluorescence analysis was also coincided with the observed one above (Figs. S10 and S11). Moreover, the role of Tan IIA on forebrain differentiation was explored at days 30 and 50. The mRNA of PAX6 was remarkably up-regulated at day 30, indicating its role in promoting cell differentiation in forebrain cell populations (Fig. 5B). However, no significant variations in PAX6 and FOXG1 expression were observed between the 2 groups at day 50 (Fig. 5D). All the results above showed that Tan IIA promoted hypoxia-mediated neurogenesis in cortical organoids, accompanied by efficient neuronal induction and neural progenitor differentiation.

### The effects of Tan IIA on neural maturation

In addition to investigating the efficacy on early neural differentiation and proliferation, Tan IIA was subsequently evaluated for their potential in the neural maturity of hypoxia-exposed organoids on chip (Fig. 6A). Following cortical neurogenesis, organoids continuously expand over long-term culture and mature, characterized by the generation of astrocytes from the same progenitor cells that gave rise to neurons. Astrocytes have been reported to play a key role in the homeostasis and function of the CNS. They may be potential cell targets for neuroprotective strategies as they are involved in the pathophysiology and the response to a number of neuropathological conditions [34,35]. Thus, we next explored the impacts of Tan IIA on astrocytes in the cortical organoids-on-a-chip under physiological hypoxia. To identify the differentiated characterization of astrocytes, the gene expression of the astrocyte activation marker GFAP was examined by qRT-PCR (Fig. 6B). The result displayed remarkably higher expression within the Tan IIA-treated organoids than in the control. Besides mRNA expression, alterations in the GFAP protein level were analyzed by immunohistochemical staining and quantitative data. As shown in Fig. 6C and D, Tan IIA treatment resulted in a slightly higher proportion of astrocytes, indicating that Tan IIA triggers a greater level of astrocyte differentiation.

Enhanced synaptogenesis and synaptic remodeling assist in CNS development as well as systematic regulation [36,37]; we thus performed gene expression analyses on organoids via qRT-PCR to determine the mRNA levels of important genes for synaptic functions. We found that Tan IIA-treated organoids showed up-regulation of synaptic related genes, such as SYN1, DBN1, DLG4, and TCF7L2 at day 50 (Fig. 6E to H). We further confirmed these results using immunohistochemical staining, which revealed the formation of intricate neuronal networks among organoid slices in both groups with or without Tan IIA. Synaptogenesis was assessed by measuring synaptic puncta density, determined by the actin binding protein drebrin and postsynaptic αPSD95. In comparison with the control, increases in synaptic puncta density was detected in Tan IIA-exposed organoids at day 50 (Fig. 6I). In addition, TCF7L2+ cells in cortical organoids that underwent treatment with Tan IIA were assessed by immunofluorescence staining (Fig. 6I), and the quantitative analysis identified that Tan IIA-exposed organoids displayed higher TCF7L2 expression than the control (Fig. S11). Together, these results suggest a positive role for Tan IIA in synaptic remodeling and maturation within hypoxia-induced cortical organoids.

## Conclusion

We fabricated an engineered cortical organoid-on-a-chip which could simulate hypoxic environments in vivo and, on this basis, made it possible to explore the efficacy of Tan IIA on human early-gestational cortex neurodevelopment. In comparison to conventional culture systems, the engineered cortical organoids with physiological hypoxia displayed induced neural differentiation and corticogenesis, as well as functional neuronal network. The results consequently indicated the potential of hypoxic culture conditions to drive the differentiation of cortical organoids toward a more in vivo-like model. Building on this, we explored the role of Tan IIA in specific types of cells within cortical organoids. Tan IIA exhibited the ability to promote neuronal activities, as evidenced by enhanced neuronal differentiation and neural functional network maturation. These data suggested that multiple aspects of cortical organoid functional characteristics were strongly impacted by the presence of Tan IIA, implicating that Tan IIA satisfies the nature of the drug development strategy for neurodevelopmental disorders and neurodegenerative diseases. Taken together, the engineered hiPSC-derived cortical organoids on the microfluidic chip with an in vivo-like microenvironment reproduce embryonic cortical differentiation. This novel approach enables the systematic screening of Chinese medicine that promotes embryonic brain development, a challenging task on human embryonic tissues, promising to facilitate the international development and application of Chinese medicine.

## Materials and Methods

### Fabrication and characterization of the microfluidic chip

Cortical organoid-on-a-chip was designed by C4D software and fabricated by a projection micro stereolithography 3D printing system with an accuracy of 2 μm. The chip was prepared from a light-curing resin built on functional acrylates, photo-initiators, and cross-linkers, and was divided into 2 parts: a top layer and a bottom layer. The bottom layer was composed of 4 chambers containing micropillars (height: 0.5 mm, diameter: 1 mm, gaps: 50 μm). Channels (height: 1 mm) for cell injection and fluid flow were present in the top layer. The internal structure of the microfluidic chip was characterized using scanning electron microscopy.

### HiPSC culture and maintenance

As described previously [38], hiPSCs (a gift from Prof. Lijian Hui) were cultivated in mTeSR1 on Matrigel-precoated 6-well plates. Cells were digested into small colonies by Accutase (Sigma) when the hiPSC fusion exceeded 80% and passaged at a 1:5 ratio. Cells were subsequently maintained for 1 h in mTeSR1 media with the ROCK inhibitor Y27632 (10 μM). Finally, cells were cultivated in mTeSR1 medium without Y27632 and replaced daily.

### Cortical organoid formation

To form cortical organoids, single hiPSCs were seeded on chip and maintained in knockout serum replacement (KSR) medium with 10 μM Y27632 and 4 ng ml^−1^ basic fibroblast growth factor for 1 to 2 d. The following compounds were incorporated in the KSR medium: 80% advanced Dulbecco’s modified Eagle medium/nutrient mixture F-12 (Gibco), 20% KSR (Gibco), 1% MEM-Eagle with nonessential amino acids (Invitrogen), 1% GlutaMAX (Invitrogen), and 1% penicillin-streptomycin (Sigma). EBs were cultivated in the KSR medium by addition of dorsomorphin (Selleck) (100 ng ml^−1^) and SB431542 (100 ng ml^−1^) for another 3 d and then in KSR medium from days 4 to 8. KSR medium with basic fibroblast growth factor (4 ng ml^−1^) was added for another 3 d. From day 12 to the last, medium was changed into neural induction medium, which contained 50% neurobasal medium (Gibco), 50% advanced Dulbecco’s modified Eagle medium/nutrient mixture F-12, 0.5% MEM-Eagle with nonessential amino acids, 1% penicillin-streptomycin, 1% B27 supplement (Gibco), 1% GlutaMAX, and 1 μg ml^−1^ heparin (Sigma).

### Formation of cortical organoids with physiological hypoxia

To generate organoids with physiological hypoxia, human cortical organoids (day 1) derived from hiPSCs were maintained in a hypoxic incubator that contained 90% N_2_, 5% CO_2_, and 5% O_2_ for 30 or 50 d and then collected for analysis at the desired time points.

### Exposure of cortical organoids to Tan IIA

At 20 or 40 d of in vitro differentiation under hypoxia, cortical organoids were exposed to 1 μM Tan IIA (Sigma, T4952) for 72 h. Then, organoids were cultivated for another 7 d without Tan IIA. Finally, cortical organoids were prepared for subsequent biological analysis at the desired time points.

### qRT-PCR

In brief, RNAiso Plus was applied to extracted total mRNA from organoids. mRNA concentration was measured and adjusted to 200 ng μl^−1^. PrimeScript RT Master Mix (Takara) was applied for reverse transcription in the samples. Ex Taq DNA polymerase (Takara) was employed to amplify cDNA. Table S1 lists the primers utilized in this investigation.

### Flow cytometry

3D tissues were first maintained in neural induction medium with Y27632 (5 μM) for 1 h. Then, they were digested after incubation with 0.125% trypsin for 10 min and filtered through cell strainers (40 μm). Then, cells were prepared and analyzed by the LIVE/DEAD Fixable Near-IR Dead Cell Stain Kit (Invitrogen). The analysis has been performed in accordance with the manufacturer’s instruction. The experiments were conducted using C6 flow cytometry (BD Biosciences), and the results were processed through FlowJo Software.

### Calcium imaging

The cortical organoids were first cultured in Matrigel-coated confocal petri dishes for 1 d, and organoids were observed to rapidly attach to the dish, facilitating calcium imaging. Calcium imaging was assessed following Fluo-4 direct calcium assay kits (Invitrogen) and was observed by a confocal laser scanning system.

### Tissue cryosection and immunohistochemistry

Cortical organoids were incubated with sucrose (30%) at 4 °C overnight after being fixed in 4% paraformaldehyde for 20 min and then immersed in optimal cutting temperature compound (Sakura) for cryosections. The sections were cleaned before being permeabilized for 5 min at room temperature with 0.2% Triton X-100 and were incubated with primary antibodies at 4 °C overnight after being blocked with 10% blocking serum (Solarbio, SL1). The primary antibodies included NESTIN (mouse, Santa Cruz Biotechnology, sc-20978, 1:400), SOX2 (rabbit, Cell Signaling Technology, 3579, 1:400), TUJ1 (mouse, BioLegend, 801201, 1:500), CTIP2 (rat, Abcam ab18465, 1:500), TBR1 (rabbit, Abcam, ab31940, 1:200), GFAP (mouse, Beyotime, AG259-1, 1:200), SATB2 (rabbit, Proteintech, 21307-1-AP, 1:500), HIF-1α (mouse, Santa Cruz Biotechnology, sc-13515, 1:200), and GABA (rabbit, Sigma, A2052, 1:400). Then, samples were incubated for 1 h with the secondary antibody (Invitrogen, 1:1,000) before washing with PBS, and nuclei were counterstained with DAPI (Sigma) for another 5 min and subsequently inspected by a confocal microscope.

### Statistical analysis

Data are expressed as the means ± SD. The data were analyzed by the Student *t* test. Significance levels were indicated as follows: *P < 0.05; **P < 0.01; ***P < 0.001; ****P < 0.0001. Sample sizes were indicated in the figure legends. Immunostaining images were quantified with Image-Pro Plus 6.0. In addition, the data were processed with Excel, Origin8, and GraphPad Prism 5.

## Data Availability

Supplementary information accompanying this paper is available.

## References

[1] Juric-Sekhar G, Hevner RF. Malformations of cerebral cortex development: Molecules and mechanisms. Annu Rev Pathol. 2019;14:293–318. 30677308 10.1146/annurev-pathmechdis-012418-012927 PMC6938687

[2] Klingler E, Francis F, Jabaudon D, Cappello S. Mapping the molecular and cellular complexity of cortical malformations. Science. 2021;371(6527):eaba4517. 33479124 10.1126/science.aba4517

[3] Paulsen B, Velasco S, Kedaigle AJ, Pigoni M, Quadrato G, Deo AJ, Adiconis X, Uzquiano A, Sartore R, Yang SM, et al. Autism genes converge on asynchronous development of shared neuron classes. Nature. 2022;602(7896):268–273. 35110736 10.1038/s41586-021-04358-6 PMC8852827

[4] Wang J, Qiao H, Wang Z, Zhao W, Chen T, Li B, Zhu L, Chen S, Gu L, Wu Y, et al. Rationally design and acoustically assemble human cerebral cortex-like microtissues from hiPSC-derived neural progenitors and neurons. Adv Mater. 2023;35(32):e2210631. 37170683 10.1002/adma.202210631

[5] Ku T, Ren Z, Yang R, Liu QS, Sang N, Faiola F, Zhou Q, Jiang G. Abnormal neural differentiation in response to graphene quantum dots through histone modification interference. Environ Int. 2022;170: Article 107572. 36228552 10.1016/j.envint.2022.107572

[6] Liu J, Gao D, Hu D, Lan S, Liu Y, Zheng H, Yuan Z, Sheng Z. Delivery of biomimetic liposomes via meningeal lymphatic vessels route for targeted therapy of Parkinson’s disease. Research (Wash D C). 2023;6. 10.34133/research.0030 PMC10076012 37040500

[7] Velasco S, Kedaigle AJ, Simmons SK, Nash A, Rocha M, Quadrato G, Paulsen B, Nguyen L, Adiconis X, Regev A, et al. Individual brain organoids reproducibly form cell diversity of the human cerebral cortex. Nature. 2019;570(7762):523–527. 31168097 10.1038/s41586-019-1289-x PMC6906116

[8] Qian X, Su Y, Adam CD, Deutschmann AU, Pather SR, Goldberg EM, Su K, Li S, Lu L, Jacob F, et al. Sliced human cortical organoids for modeling distinct cortical layer formation. Cell Stem Cell. 2020;26(5):766–781.e769. 10.1016/j.stem.2020.02.002. 32142682 PMC7366517

[9] Pașca AM, Park JY, Shin HW, Qi Q, Revah O, Krasnoff R, O'Hara R, Willsey AJ, Palmer TD, Pașca SP. Human 3D cellular model of hypoxic brain injury of prematurity. Nat Med. 2019;25(5):784–791. 31061540 10.1038/s41591-019-0436-0 PMC7020938

[10] Zhu Y, Zhang X, Sun L, Wang Y, Zhao Y. Engineering human brain assembloids by microfluidics. Adv Mater. 2023;35(14): Article e2210083. 36634089 10.1002/adma.202210083

[11] Jung GA, Kim JA, Park HW, Lee H, Chang MS, Cho KO, Song BW, Kim HJ, Kwon YK, Oh IH. Induction of Nanog in neural progenitor cells for adaptive regeneration of ischemic brain. Exp Mol Med. 2022;54(11):1955–1966. 36376495 10.1038/s12276-022-00880-3 PMC9722910

[12] Cheng L, Hu W, Qiu B, Zhao J, Yu Y, Guan W, Wang M, Yang W, Pei G. Generation of neural progenitor cells by chemical cocktails and hypoxia. Cell Res. 2014;24(6):665–679. 24638034 10.1038/cr.2014.32 PMC4042166

[13] Xie Y, Zhang J, Lin Y, Gaeta X, Meng X, Wisidagama DRR, Cinkornpumin J, Koehler CM, Malone CS, Teitell MA, et al. Defining the role of oxygen tension in human neural progenitor fate. Stem Cell Reports. 2014;3(5):743–757. 25418722 10.1016/j.stemcr.2014.09.021 PMC4235163

[14] Li G, Liu J, Guan Y, Ji X. The role of hypoxia in stem cell regulation of the central nervous system: From embryonic development to adult proliferation. CNS Neurosci Ther. 2021;27(12):1446–1457. 34817133 10.1111/cns.13754 PMC8611781

[15] Valverde MG, Mille LS, Figler KP, Cervantes E, Li VY, Bonventre JV, Masereeuw R, Zhang YS. Biomimetic models of the glomerulus. Nat Rev Nephrol. 2022;18(4):241–257. 35064233 10.1038/s41581-021-00528-x PMC9949601

[16] Tang Z, Kong N, Zhang X, Liu Y, Hu P, Mou S, Liljeström P, Shi J, Tan W, Kim JS, et al. A materials-science perspective on tackling COVID-19. Nat Rev Mater. 2020;5(11):847–860. 33078077 10.1038/s41578-020-00247-y PMC7556605

[17] Wang Z, Wang Y, Yan J, Zhang K, Lin F, Xiang L, Deng L, Guan Z, Cui W, Zhang H. Pharmaceutical electrospinning and 3D printing scaffold design for bone regeneration. Adv Drug Deliv Rev. 2021;174:504–534. 33991588 10.1016/j.addr.2021.05.007

[18] Saiding Q, Chen X, Cui W. Programmable multicellular and spatially patterned organoids: A one-pot strategy. Matter. 2022;5:1633–1635.

[19] Chen X, Zhang YS, Zhang X, Liu C. Organ-on-a-chip platforms for accelerating the evaluation of nanomedicine. Bioact Mater. 2021;6(4):1012–1027. 33102943 10.1016/j.bioactmat.2020.09.022 PMC7566214

[20] Deng G, Zhou L, Wang B, Sun X, Zhang Q, Chen H, Wan N, Ye H, Wu X, Sun D, et al. Targeting cathepsin B by cycloastragenol enhances antitumor immunity of CD8 T cells via inhibiting MHC-I degradation. J Immunother Cancer. 2022;10(10):e004874. 36307151 10.1136/jitc-2022-004874 PMC9621195

[21] Zhou X, Saiding Q, Wang X, Wang J, Cui W, Chen X. Regulated exogenous/endogenous inflammation via “inner-outer” medicated electrospun fibers for promoting tissue reconstruction. Adv Healthc Mater. 2022;11(10): Article e2102534. 34989182 10.1002/adhm.202102534

[22] Fan N, Zhao J, Zhao W, Zhang X, Song Q, Shen Y, Shum HC, Wang Y, Rong J. Celastrol-loaded lactosylated albumin nanoparticles attenuate hepatic steatosis in non-alcoholic fatty liver disease. J Control Release. 2022;347:44–54. 35483638 10.1016/j.jconrel.2022.04.034

[23] Luan X, Zhang X, Nie M, Zhao Y. Traditional Chinese medicine integrated responsive microneedles for systemic sclerosis treatment. Research (Wash D C). 2023;2023:6. 10.34133/research.0141 PMC10204745 37228639

[24] Maione F, Piccolo M, De Vita S, Chini MG, Cristiano C, De Caro C, Lippiello P, Miniaci MC, Santamaria R, Irace C, et al. Down regulation of pro-inflammatory pathways by tanshinone IIA and cryptotanshinone in a non-genetic mouse model of Alzheimer’s disease. Pharmacol Res. 2018;129:482–490. 29158049 10.1016/j.phrs.2017.11.018

[25] Liu X, Ye M, An C, Pan L, Ji L. The effect of cationic albumin-conjugated PEGylated tanshinone IIA nanoparticles on neuronal signal pathways and neuroprotection in cerebral ischemia. Biomaterials. 2013;34(28):6893–6905. 23768781 10.1016/j.biomaterials.2013.05.021

[26] Subedi L, Gaire BP. Tanshinone IIA: A phytochemical as a promising drug candidate for neurodegenerative diseases. Pharmacol Res. 2021;169: Article 105661. 33971269 10.1016/j.phrs.2021.105661

[27] Tang XY, Xu L, Wang J, Hong Y, Wang Y, Zhu Q, Wang D, Zhang XY, Liu CY, Fang KH, et al. DSCAM/PAK1 pathway suppression reverses neurogenesis deficits in iPSC-derived cerebral organoids from patients with down syndrome. J Clin Invest. 2021;131(12):e135763. 33945512 10.1172/JCI135763 PMC8203468

[28] Harris JA, Mihalas S, Hirokawa KE, Whitesell JD, Choi H, Bernard A, Bohn P, Caldejon S, Casal L, Cho A, et al. Hierarchical organization of cortical and thalamic connectivity. Nature. 2019;575(7781):195–202. 31666704 10.1038/s41586-019-1716-z PMC8433044

[29] Pan Z, Ma G, Kong L, Du G. Hypoxia-inducible factor-1: Regulatory mechanisms and drug development in stroke. Pharmacol Res. 2021;170: Article 105742. 34182129 10.1016/j.phrs.2021.105742

[30] Cadwell CR, Bhaduri A, Mostajo-Radji MA, Keefe MG, Nowakowski TJ. Development and arealization of the cerebral cortex. Neuron. 2019;103(6):980–1004. 31557462 10.1016/j.neuron.2019.07.009 PMC9245854

[31] Libedinsky C. Comparing representations and computations in single neurons versus neural networks. Trends Cogn Sci. 2023;27(6):517–527. 37005114 10.1016/j.tics.2023.03.002

[32] Molnár Z, Luhmann HJ, Kanold PO. Transient cortical circuits match spontaneous and sensory-driven activity during development. Science. 2020;370(6514):eabb2153. 33060328 10.1126/science.abb2153 PMC8050953

[33] Chu F, Tan R, Wang X, Zhou X, Ma R, Ma X, Li Y, Liu R, Zhang C, Liu X, et al. Transcranial magneto-acoustic stimulation attenuates synaptic plasticity impairment through the activation of Piezo1 in Alzheimer’s disease mouse model. Research (Wash D C). 2023;6:0130. 37223482 10.34133/research.0130 PMC10202414

[34] Szebényi K, Wenger LMD, Sun Y, Dunn AWE, Limegrover CA, Gibbons GM, Conci E, Paulsen O, Mierau SB, Balmus G, et al. Human ALS/FTD brain organoid slice cultures display distinct early astrocyte and targetable neuronal pathology. Nat Neurosci. 2021;24(11):1542–1554. 34675437 10.1038/s41593-021-00923-4 PMC8553627

[35] Lee HG, Wheeler MA, Quintana FJ. Function and therapeutic value of astrocytes in neurological diseases. Nat Rev Drug Discov. 2022;21(5):339–358. 35173313 10.1038/s41573-022-00390-x PMC9081171

[36] Adams JW, Negraes PD, Truong J, Tran T, Szeto RA, Guerra BS, Herai RH, Teodorof-Diedrich C, Spector SA, Del Campo M, et al. Impact of alcohol exposure on neural development and network formation in human cortical organoids. Mol Psychiatry. 2023;28(4):1571–1584. 36385168 10.1038/s41380-022-01862-7 PMC10208963

[37] Song W, Li Q, Wang T, Li Y, Fan T, Zhang J, Wang Q, Pan J, Dong Q, Sun ZS, et al. Putative complement control protein CSMD3 dysfunction impairs synaptogenesis and induces neurodevelopmental disorders. Brain Behav Immun. 2022;102:237–250. 35245678 10.1016/j.bbi.2022.02.027

[38] Zhu Y, Sun L, Fu X, Liu J, Liang Z, Tan H, Li W, Zhao Y. Engineering microcapsules to construct vascularized human brain organoids. Chem Eng J. 2021;424(7467):130427.

