# Supplementary material for: Cortical Organoid-on-a-Chip with Physiological Hypoxia for Investigating Tanshinone IIA-Induced Neural Differentiation
Source: Research (Wash D C). 2023 Nov 22;6:0273. doi: 10.34133/research.0273 (PMC10907018; doi:10.34133/research.0273)
Supplement: Supplementary 1 — Figs. S1 to S11 Table S1 [file research.0273.f1.docx]

**Supplementary Materials**

**Cortical organoid-on-a-chip with physiological hypoxia for investigating tanshinone ⅡA-induced neural differentiation**

*Yue Zhi, Yujuan Zhu, Jinglin Wang, Junqi Zhao, Yuanjin Zhao**


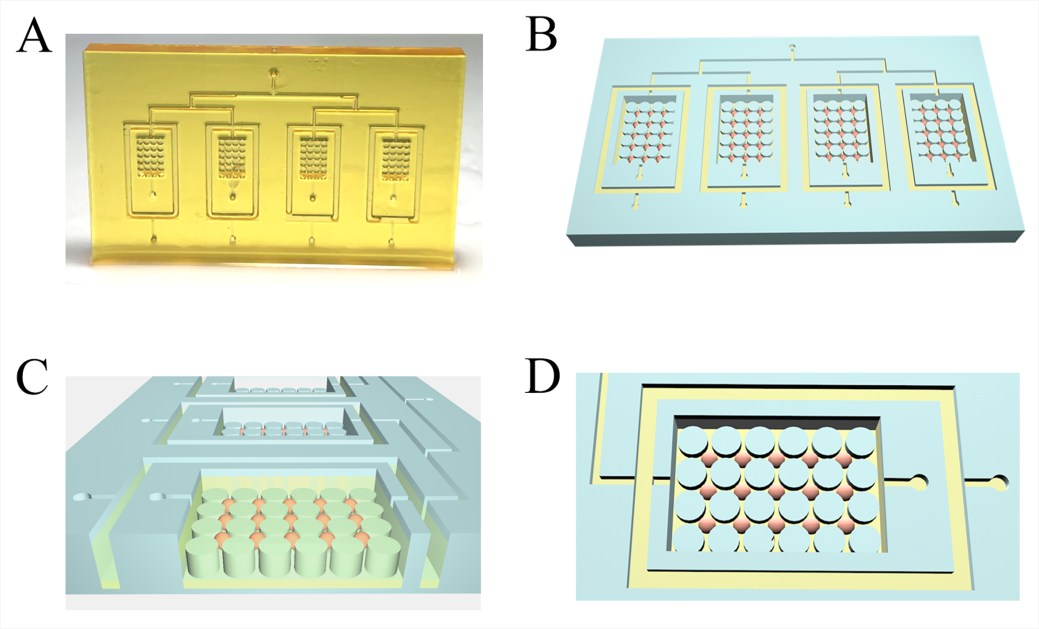


**Figure S1 Bright field diagram and schematic diagram.** (a) Bright field image of the 3D printed microfluidic chip. (b-d) Schematic illustrations of the microfluidic chip. The chip consisted of four independent chambers with micropillar arrays.


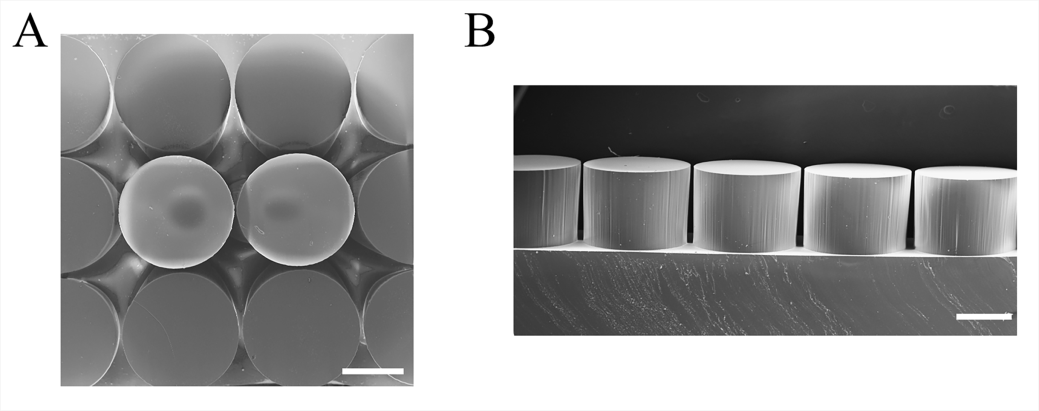


**Figure S2 SEM images of the microfluidic chip.** (a-b) SEM images of top view (a) and cross-sectional view (b) of micropillars within the microfluidic chip. Scale bars, 500 μm.


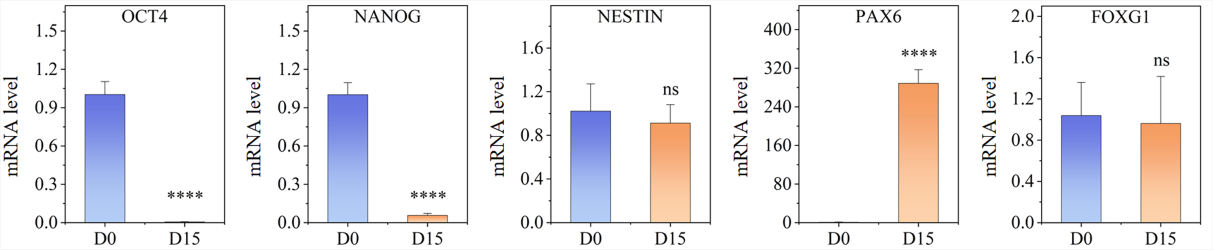


**Figure S3 qRT-PCR analysis.** mRNA expression of pluripotency markers (OCT4, NANOG) and neural markers (NESTIN, PAX6 and FOXG1) at 0 and 10 days of differentiation (n = 3). Three independent experiments had been performed. Data are shown as mean ± SD. ns, not significant; *****p* < 0.0001.


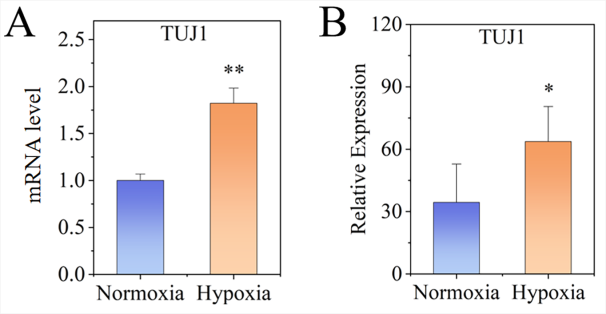


**Figure S4 The expression of neuronal marker TUJ1.** (a) The relative mRNA expression of TUJ1 in hypoxia- or normoxia-exposed cortical organoids at day 30 was identified by qRT-PCR (n = 3). (b) The fluorescence intensity of TUJ1 was quantified (n = 6). All data are the means of three replicates ± SD. The data were analyzed using the Student’s *t*-test. (**p* < 0.05; ***p* < 0.01).


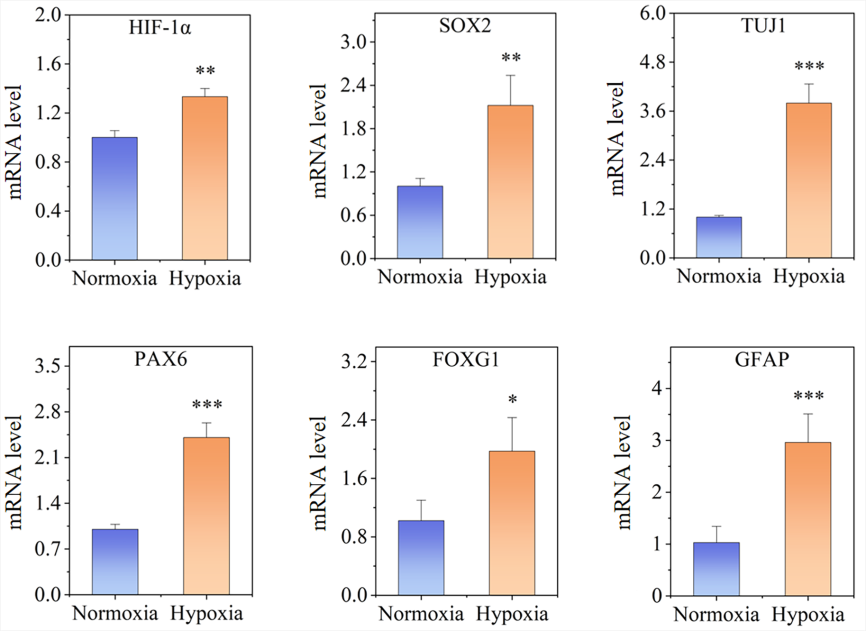


**Figure S5 qRT-PCR analysis.** The mRNA expression of related markers for neural differentiation and maturation cortical organoids on chip after 50 days of differentiation (n = 3). All data are the means of three replicates ± SD. The data were analyzed using the Student’s *t*-test. (**p* < 0.05; ***p* < 0.01; ****p* < 0.001; *****p* < 0.0001).


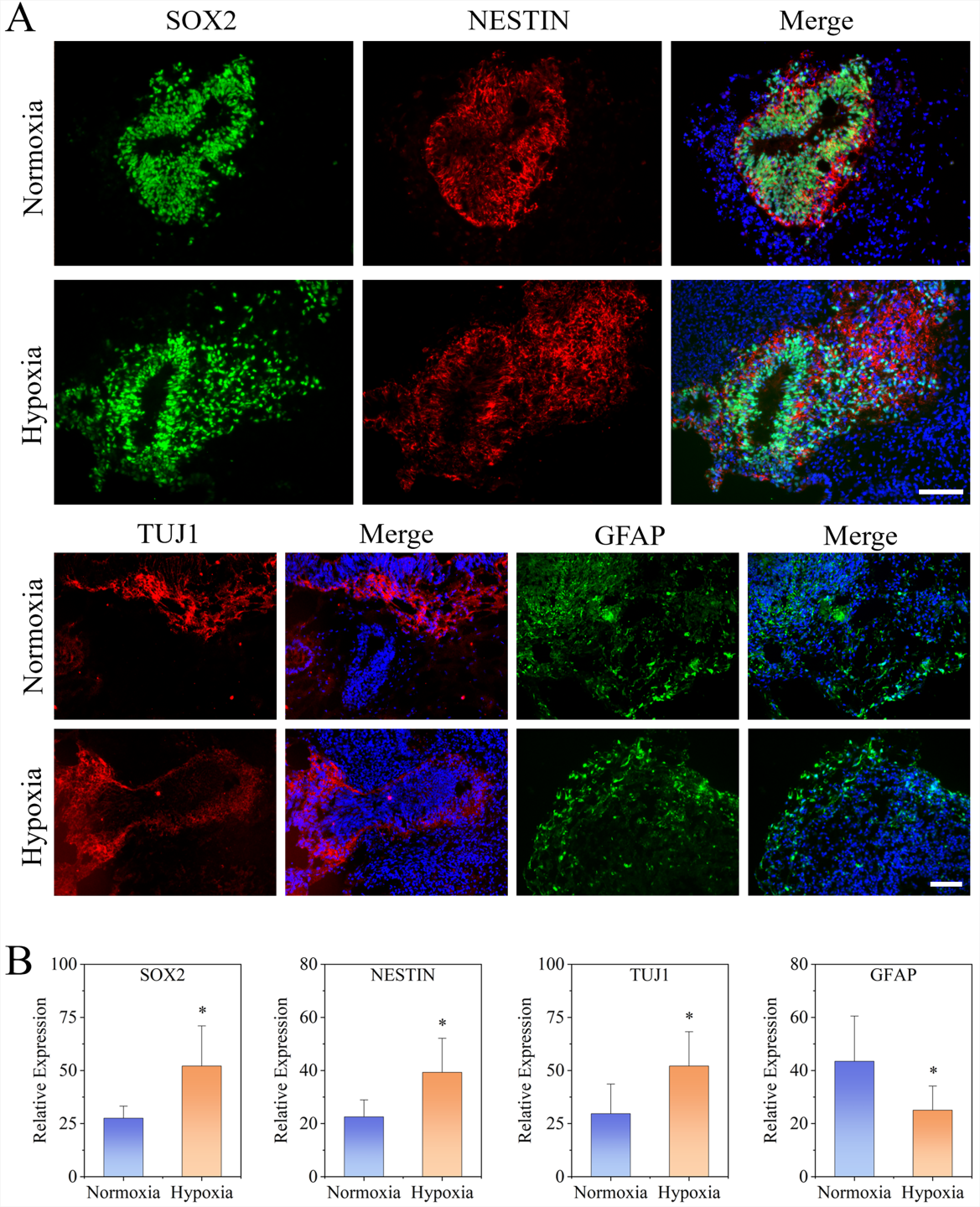


**Figure S6 Immunofluorescence staining and corresponding quantification.** (a-b) Immunostaining images of key developmental markers in organoids with hypoxia or normoxia at day 50 were shown. All data are the means of six replicates ± SD. The data were analyzed using the Student’s *t*-test. (**p* < 0.05). Scale bars, 100 μm.


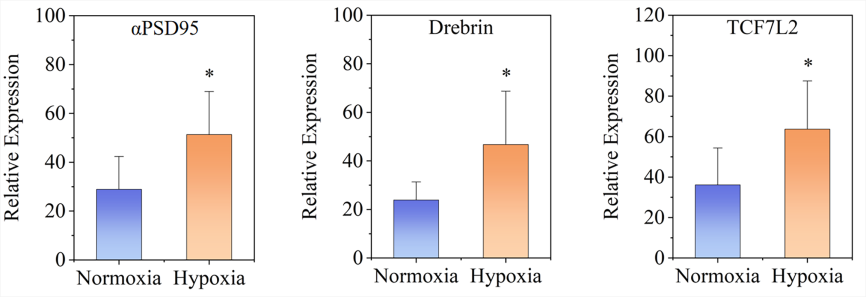


**Figure S7 Quantitative analysis.** Quantitative expression of synaptic markers in cortical organoids under hypoxic and normoxic conditions at day 50 (n = 6).All data are the means of six replicates ± SD. The data were analyzed using the Student’s *t*-test. (**p* < 0.05).


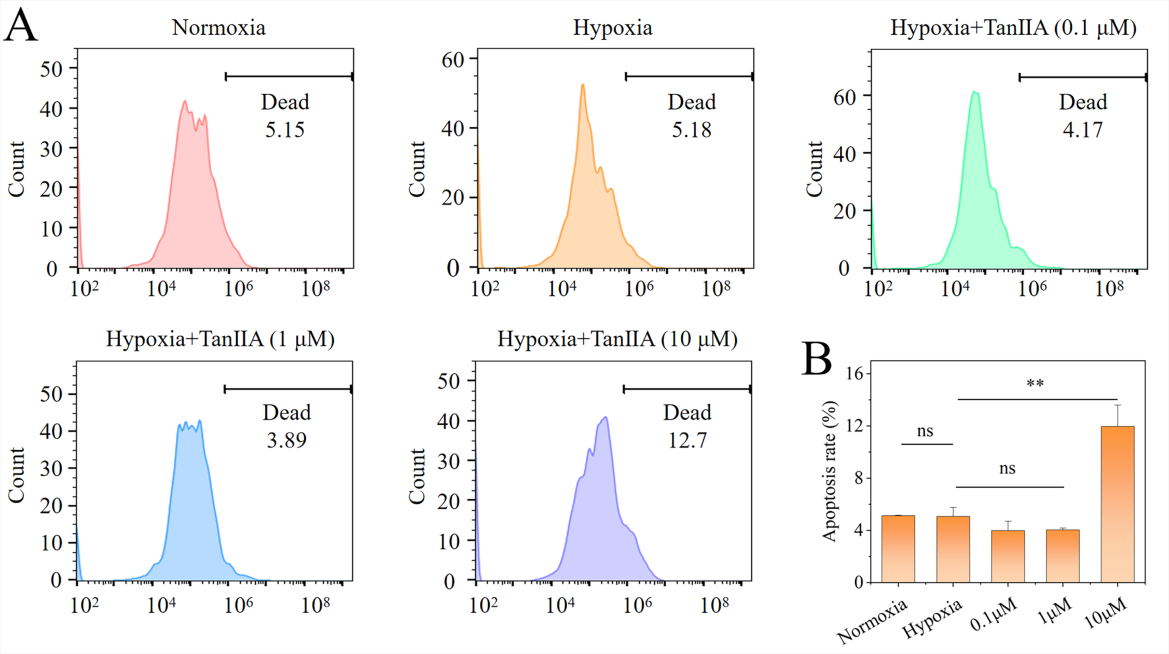


**Figure S8 Detection by flow cytometry.** (a) FACS analysis of live and dead cells in cortical organoids with different treatment. (b) Quantitative analysis of apoptosis in cortical organoids with different treatment using flow cytometry (n = 3). All data are the means of three replicates ± SD. The data were analyzed using the Student’s *t*-test. (ns, not significant; ***p* < 0.01).


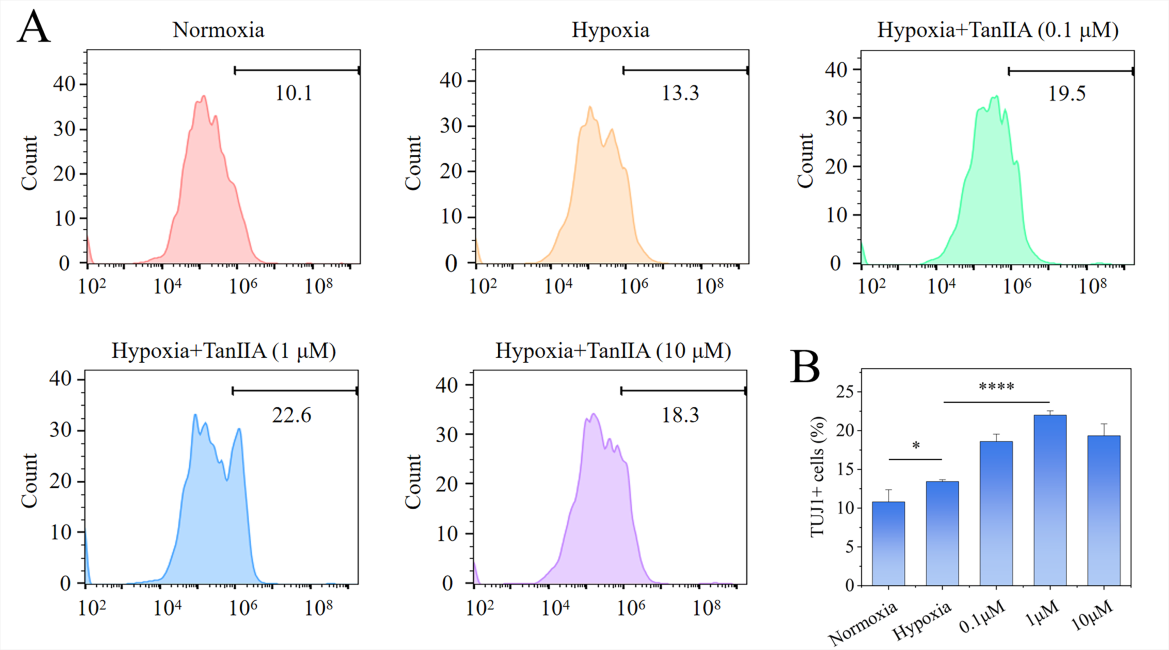


**Figure S9 Detection by flow cytometry.** (a) FACS analysis of TUJ1+ cells in cortical organoids with different treatment. (b) Quantitative analysis of TUJ1+ cells in cortical organoids with different treatment using flow cytometry (n = 3). All data are the means of three replicates ± SD. The data were analyzed using the Student’s *t*-test. (**p* < 0.05; *****p* < 0.0001).


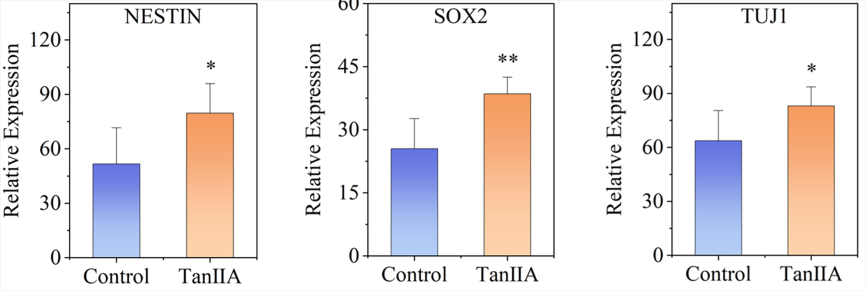


**Figure S10 Quantitative analysis.** Quantitative expression of neuronal markers in cortical organoids treated with and without Tan IIA under hypoxia environment at day 30 (n = 6). All data are the means of six replicates ± SD. The data were analyzed using the Student’s *t*-test. (**p* < 0.05; ***p* < 0.01).


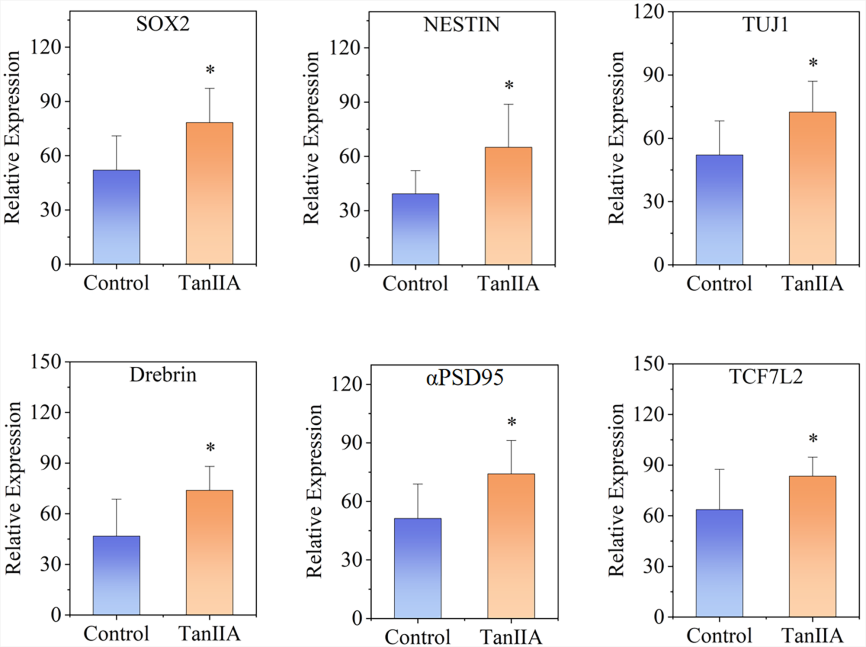


**Figure S11 Quantitative analysis.** Quantitative expression of different types of cells in cortical organoids treated with and without Tan IIA under hypoxia environment at day 50 (n = 6). All data are the means of six replicates ± SD. The data were analyzed using the Student’s *t*-test. (**p* < 0.05).

**Table S1 Target gene primer sequences**

| Gene |  | Primers |
| --- | --- | --- |
| SOX2 | F | TACAGCATGTCCTACTCGCAG |
|  | R | GAGGAAGAGGTAACCACAGGG |
| PAX2 | F | TGTCAGCAAAATCCTGGGCAG |
|  | R | GTCGGGTTCTGTCGTTTGTATT |
| PAX6 | F | TGGGCAGGTATTACGAGACTG |
|  | R | ACTCCCGCTTATACTGGGCTA |
| TUJ1 | F | CTCAGGGGCCTTTGGACATC |
|  | R | CAGGCAGTCGCAGTTTTCAC |
| FOXG1 | F | GAGCGACGACGTGTTCATC |
|  | R | GCCGTTGTAACTCAAAGTGCTG |
| GFAP | F | AGGTCCATGTGGAGCTTGAC |
|  | R | GCCATTGCCTCATACTGCGT |
| HIF-1α | F | ATCCATGTGACCATGAGGAAATG |
|  | R | TCGGCTAGTTAGGGTACACTTC |
| GAPDH | F | AGGTCGGTGTGAACGGATTTG |
|  | R | TGTAGACCATGTAGTTGAGGTCA |
